# Laboratory methods to improve SELDI peak detection and quantitation

**DOI:** 10.1186/1477-5956-5-9

**Published:** 2007-07-02

**Authors:** Dominique Rollin, Toni Whistler, Suzanne D Vernon

**Affiliations:** 1 Chronic Viral Diseases Branch, Division of Viral and Rickettsial Diseases, Centers for Disease Control and Prevention, 1600 Clifton Rd, MS-G41 Atlanta, Georgia 30333, USA

## Abstract

**Background:**

Protein profiling with surface-enhanced laser desorption-ionisation time-of-flight mass spectrometry (SELDI-TOF MS) is a promising approach for biomarker discovery. Some candidate biomarkers have been identified using SELDI-TOF, but validation of these can be challenging because of technical parameters that effect reproducibility. Here we describe steps to improve the reproducibility of peak detection.

**Methods:**

SELDI-TOF mass spectrometry was performed using a system manufactured by Ciphergen Biosystems along with their ProteinChip System. Serum from 10 donors was pooled and used for all experiments. Serum was fractionated with Expression Difference Mapping kit-Serum Fractionation from the same company and applied to three different ProteinChips. The fractionations were run over a one month period to examine the contribution of sample batch and time to peak detection variability. Spectra were processed and peaks detected using the Ciphergen Express software and variance measured.

**Results:**

Experimental parameters specific to the serum fraction and ProteinChip, including spot protocols (laser intensity and detector sensitivity) were optimized to decrease peak detection variance. Optimal instrument settings, regular calibration along with controlled sample handling and processing nearly doubled the number of peaks detected and decreased intensity variance.

**Conclusion:**

This report assesses the variation across fractionated sera processed over a one-month period. The optimizations reported decreased the variance and increased the number of peaks detected.

## Background

The SELDI-TOF mass spectroscopy platform was designed for high-throughput protein profiling and biomarker discovery. The resolution of SELDI-TOF has been improved by incorporating fractionation and a variety of affinity capture techniques [1,2]. Still there are sources of technical and biologic variation which make reproducing and validating potential biomarkers challenging [3-6]. Further refinements to the technique are necessary to ensure that the variability in mass spectra is due to biology and to minimize systematic biases from non-disease associated factors [7-9].

Validation of disease biomarkers relies on optimized and reproducible laboratory methods. The automation of the SELDI-TOF platform and the standardization of parameters for analysis have resulted in good intra- and inter-laboratory correlation and relatively reproducible results [2,3,8]. However, there is still a need to identify the sources of variation and determine how to reduce the variation to make the SELDI-TOF platform reliable and reproducible. We examined some the front-end steps, including sample handling and preparation that occur during SELDI-TOF. Fine-tune adjustments of laser intensity and detector sensitivity for each chip type and each fraction coupled with spot-to-spot correction increased peak detection and significantly decreased the intensity coefficient of variation (CV). These further refinements to the SELDI-TOF platform will enhance biomarker identification and validation efforts.

## Results

We identified the ProteinChips and fractions with the most informative mass spectra (Table 1), for use in our comparisons. Generally, Fraction 2 (F2) was very sparse in mass peaks on all chips and F5 had a small number of peaks, not consistently detected and generally of a lower quality. Spectra included in analyses had normalization factors <2 standard deviations (SD) from the mean. Spectra with normalization factors greater than the mean ± 2SD tended to be poor quality spectra, either with heavy matrix noise and no peaks, or poor peak intensities. This was chosen as the cut-off value for spectral exclusion. The minimum peak threshold setting for peak detection was set at 80% for both experiments, insuring the minor variations in peak patterns seen in Exp 1 were kept in the datasets.

**Table 1:** Fractions and ProteinChips considered having sufficient complexity for the analysis.

ProteinChip	Fractions used in analysis				
IMAC	F1	F3	F4		**F6**
H50	F1	**F3**	**F4**		F6
CM10-LS	F1	F3	F4	F5	**F6**
CM10-HS		**F3**	F4		

Bold – denotes fractions to be highly variable across batches.

Table 2 presents results comparing the effect of experimental conditions on spectra quality across all batches. These included laser intensity and detector sensitivity adjustments to spot protocols for each fraction, automated applications and defined drying steps. Fewer spectra were excluded in Exp 2 with several ProteinChip/Fraction combinations having no excluded spectra. The largest impact, as assessed by number of spectra removed, appears to be on the CM10 ProteinChip surface (Table 2). Peak selection criteria requested that each peak needed to be present in 80% of spectra to be included in analysis. The number of peaks detected almost doubled in Exp 2 and the CVs for peak intensities improved substantially (Table 2). The quality of the peaks did not improve substantially in term of signal-to-noise ratio (S/N) and resolution, but the intensity of the peaks globally increased, and this contributed probably to better peak detection. The weak cation exchange ProteinChip (CM10), using low stringency (LS) buffers (CM10-LS), under Exp 2 conditions appeared to give the least variant, most complex spectra of all chip surfaces, as determined by number of peaks detected and range of peak intensity CVs (Table 2). We have used peak number as a reflection of spectral complexity. Other parameters can be used to define this, such as S/N ratio, signal height to valley depth.

**Table 2:** Summary table of results comparing Exp.1 and Exp.2 showing improvement in peak detection and peak intensity variation following optimization of protocols.

		Experiment 1					Experiment 2				
		
Protein Chip	Fraction	No. Spectra excluded (Total 18)	No. Peaks	Averaged CV Peak Intensity (%)	CV Range	No. Peaks (%) statistically different across batches (p < 0.01)^a^	No. Spectra excluded (Total 18)	No. Peaks	Averaged CV Peak Intensity (%)	CV Range	No. Peaks statistically (%) different across batches (p < 0.01)^a^
	F1	5	16	31.7	12–127	0	1	28	20.9	8–36	2 (7)
	F3	1	11	31.2	15–75	0	0	21	31.4	12–52	2 (10)
IMAC	F4	3	7	44.7	29–61	1 (14)	0	20	26.0	14–45	2 (10)
	F6	3	21	27.0	15–39	0	7	27	22.6	11–49	9 (33)
	LS-F1	4	22	31.0	15–50	0	1	23	18.1	11–41	1 (4)
	LS-F3	6	5	41.5	25–48	0	0	23	26.4	13–40	3 (13)
	LS-F4	1	13	37.4	18–55	6 (46)	1	26	14.7	8–30	0
CM10	LS-F5	4	9	30.6	20–46	3 (33)	0	27	16.4	8–30	1 (4)
	LS-F6	3	13	29.4	18–44	0	2	30	25.4	8–50	11 (37)
	HS-F3	6	3	66.5	53–85	0^b^	1	15	49.2	27–148	9 (60)
	HS-F4	6	5	48.7	19–72	0	3	14	30.6	10–46	0
	F1	5	6	67.5	40–129	0	0	16	32.7	15–66	2 (13)
	F3	0	13	32.4	16–50	3 (23)	1	17	22.8	15–38	5 (29)
H50	F4	2	15	33.0	15–79	2 (15)	1	21	22.5	11–53	5 (24)
	F6	2	30	23.7	13–39	1 (3)	1	35	25.0	12–55	3 (9)

^a ^This column reports the number of peaks in the ProteinChip-fraction spectra that were considered to have different peak intensities between batches as calculated by the non-parametric Kruskal-Wallis (p < 0.01), the data was bootstrapped using 2000 randomizations of the experiments to correct for multiple testing.

^b ^Analysis performed on 2 batches, not 3.

To determine the reproducibility between the 3 batches in which sera were run we looked for statistical differences in peak intensities between batches using a Kruskal-Wallis non parametric test, adjusted for multiple testing by bootstrapping. We determined the number of peaks in each batch that were statistically different (p < 0.01). The results are presented in Table 2. Four ProteinChip/Fractions in Exp 2 showed >40% of peaks determined on a single quality control (QC) serum that were statistically different across the batches (Table 2). In general, the CM10-LS fractions showed the lowest batch-to-batch variation.

## Discussion

Recent advances have been made in mass spectrometry to achieve high throughput separation and analysis of proteins and peptides with good mass accuracy and resolution. One of the most difficult challenges of the method is the reproducibility of the data over time and between laboratories. Several studies have addressed this issue looking at the impact of preanalytical variables like patient preparation [4], blood sample processing [3], and standardized analytical conditions [8,9]. The main thrust of this study was to examine the effects of spot protocol optimization on spectral quality as determined by number of peaks and signal intensity CVs on a QC sample. There are several laboratory considerations that have previously been reported and were implemented in this study. For example it is widely recommended that sample loading be handled through an automated liquid handling system [6,10], and that they be randomly loaded on the ProteinChips [11]. We used the Biomek 2000 Automation Workstation for these purposes. EAM was applied in two smaller volumes with a constant drying time before the EAM application to increase the number of peaks [6]. Reagent variability was minimized by using reagents from the same manufacturing lot [11] and the possible effects of freezing and thawing and length of time in storage [12] was considered for the serum and fractionated products.

Exp 1 details our first fractionation/profiling methods and Exp 2 our fine-tuning of the initial methodologies. EAM was initially applied manually because of concerns with pipetting such small volumes of highly volatile liquids. However, there were several advantages to automated application, including speed with which this could be performed, and consistent drying times between applications [13]. We also noticed a need to fine-tune spot protocols for each ProteinChip-fraction combination so we defined criteria based on intensity, S/N and resolution of few chosen peaks [8]. This improved the number of peaks detected and the reproducibility of the signal intensities.

By routinely performing and monitoring instrument checks we have established criteria to detect changes in instrument performance [8]. We added a spot-to-spot correction in Exp 2, but found it to have little impact on results.

Comparison on the average peak intensity CVs from Exp 1 to Exp 2 showed a marked improvement, changes of 4% to 37%. H50-F6 and IMAC-F3 did not show improvement. This indicates that the experimental parameters used in Exp 2 provided a considerable improvement in spectral quality. Comparing our results to those of other researchers is complicated due to different experimental conditions – not all sera are fractionated, CVs not calculated for the QC sample alone, different ProteinChips, m/z range differs for data collection, and/or peak selection criteria varies. Koopman et al. [14] used a fractionated QC serum to calculate intra-assay variation on 10 randomly chosen peaks (S/N > 5, m/z < 20,000) and found a mean CV = 24% for WCX-F1 (equivalent of CM10); 26% for WCX-F6 and 29% for IMAC-F1. This compared to our average CVs (from all batches) of 18, 25 and 23% respectively for the whole spectrum (as opposed to selected peaks). The QC serum serves as a good quality control for assay and ProteinChip variability and it would be helpful if all published SELDI data reported signal intensity CVs of QC sera, with the criteria used for their calculation, so more effective comparisons can be made across studies. The mass accuracies in the two experiments were in accordance with the manufacturer's specifications [9]. The optimization of the acquisition protocols at the fraction level (Exp 2) and automation of EAM application, have substantially improved the reproducibility of peak intensities.

We used a Kruskal-Wallis non-parametric test, with multiple test correction to examine the variability of peak intensities from the QC sera across the batches in which they were run. Not surprisingly we found that several ProteinChip-fraction combinations had more variability than others. Three ProteinChip-fractions had >30% of their peak intensities being statistically different at p < 0.01 level (H50-F3, CM10-LS-F6, CM10-HS-F3). This indicates that these may not be useful for biomarker discovery. This analysis in Exp 1, showed very little statistical difference between batches, which on the surface would imply better data. However, on closer examination this is not true. The spectra had fewer peaks, so less complexity and the variance was larger in each ProteinChip-fraction combination.  The analysis also pointed out good ProteinChip-fraction combinations that would perform in the most reproducible manner for biomarker discovery.

## Conclusion

In this study, we have investigated the effects of some practical factors for SELDI-TOF analysis of fractionated serum samples. The analysis of 18 spectra (3 batches of samples fractionated 2 weeks apart, 1 samples on each of 6 ProteinChips of one type) independently derived from the same pooled serum sample allowed us to investigate also the compounding effect of reproducibility over time. Reproducibility of mass intensities relies on a high level of standardization and optimization. Our study demonstrates that optimized instrument settings and calibration along with rigorous sample handling and processing can almost double peak detection and substantially decrease the peak intensity CVs. Nevertheless, we feel greater effort is needed to improve peak detection and quantitation and further investigation is needed to assess the reproducibility of the serum fractionation, looking to minimize variation when large sample numbers need to be processed.

## Methods

### Serum samples

**Figure 1:**
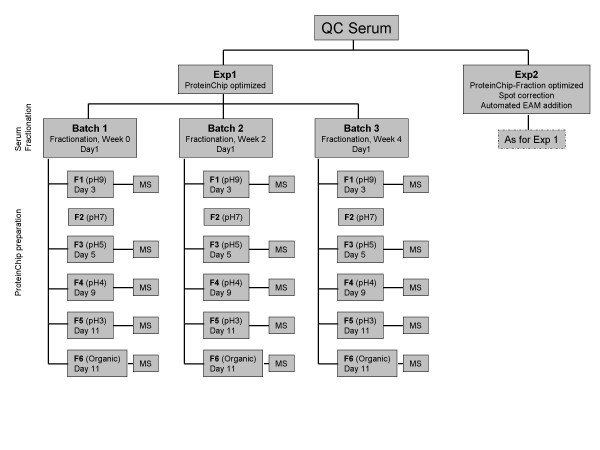
Outline of the experimental workflow for SELDI-TOF optimization. QC serum was fractionated and run as 6 replicates of fractions F1, F3, F4, and F6 on IMAC, CM10 LS and H50 ProteinChips; as 6 replicates of F3 and F4 on CM10 HS and 6 replicates of F5 on CM10 LS ProteinChips.

A QC sample was prepared by pooling serum from 10 non-fasting anonymous donors (CDC Institutional Review Board approval 1652), dispensed into single use aliquots and stored at -80°C. This QC sample was fractionated using the Biomek 2000 Automation Workstation (Beckman Coulter) and the Expression Difference Mapping Kit-Serum Fractionation according to the manufacturer (Ciphergen). Six fractions were collected and stored at -80°C for 2 to 11 days keeping storage time the same for the equivalent fractions of each sample. The fractionation procedure was repeated on the same QC sample three times with 2 weeks in between each fractionation. Each fractionation procedure representing one batch (Fig. 1).

### ProteinChips

To load the different ProteinChips, we used the correspondent Biomek 2000 Automation Workstation methods. The IMAC 30 ProteinChip was loaded with copper sulfate (Ciphergen Expression Difference Mapping kits IMAC buffer), and H50 ProteinChip (Ciphergen Expression Difference Mapping kit-H50 buffer) was washed with 50% acetonitrile before being loaded with the serum fraction. Except for these modifications, treatment of the ProteinChips included two pre-washes of 150 μl of corresponding buffer for 5 minutes each followed by addition and 1 hour incubation on a microplate shaker of 100 μl sample (10 μl serum fraction in 90 μl of buffer). Each protein chip had three 150 μl stringency washes followed by 2 quick washes with 200 μl of HPLC water. One binding-washing buffer was used for IMAC-Cu and H50 ProteinChips, and a low stringency (LS) and high stringency (HS) binding-washing buffer was used for the CM10 ProteinChip (resulting in 4 spectra for each serum fraction). After the last wash, the ProteinChips were air-dried for 20 minutes. The energy absorbing molecule (EAM) was sinapinic acid (5 mg in 200 μl acetonitrile, 200 μl of 1% trifluoroacetic acid), which was freshly prepared before a double application (1 ul each) to all ProteinChips with exactly 15 minutes drying time between each application [6]. In Exp 1 the EAM was applied by hand with a one-channel pipette. In the optimized method (Exp 2) it was applied using the Biomek 2000 robot, which dispensed 1 μL of EAM simultaneously to the eight spots of a chip. The differences between the 2 experiment protocols are outlined in Table 3. Mass analysis was performed using a PBSIIc mass spectrometer over an m/z range of 3000–30000.

**Table 3:** Differences in sample processing and analysis setting between Exp.1 and Exp.2

	EAM application	Acquisition protocol optimization	Specimen applied for optimization	Spot correction
Exp.1	Manual	Chip specific	Whole serum	No
Exp.2	Automated	Chip and Fraction specific	Appropriate serum fraction QC	Yes

### Acquisition protocols

For both studies, we optimized the spot protocols using the QC sample for the mass range between 3000 and 30000 Da. The major difference between Exp 1 and Exp 2 are the acquisition protocols. For Exp 1, spot protocols were optimized for whole serum on each different ProteinChip. To establish the optimized protocol different laser intensities and detector sensitivities were used for the collection of the spectra, and visual inspection used to assess the best spectrum. These parameters were then used in the spot protocol for experimental data acquisition. In Exp 2, spot protocols were optimized for each serum fraction-ProteinChip combination by adjusting laser intensity and detector sensitivity. The spectra collected and processed using Ciphergen Express™ software (version 3) (CE). All spectra normalized by total ion current and calibrated. Peak detection performed using with peak height of 10 and valley depth of 5. Spectral quality was assessed using 2–3 randomly selected peaks by comparing peak intensity, S/N and resolution. As Semmes et al. described [8], the laser intensity and the detector sensitivity for the spot protocol were chosen to increase the peak detection and resolution without increasing the signal to noise ratio. The optimized laser and detector sensitivity settings were used in the appropriate spot protocols. We did not change the detector voltage during the course of a study; but we changed it between the 2 experiments after optimization by DL Vary performance check as recommended by Ciphergen Biosystems. In Exp 1 the mass spectra were derived from 10 shots per transient, with a spacing of 5 between transients, acquiring a total of 130 laser shots for each spectrum. This was after 2 warming shots not included in the spectrum. In Exp 2, a total of 192 shots were collected for each spectrum, from 12 transients every 4 positions after 2 warming shots not included in the spectrum file.

### Instrument performance evaluation

QC and performance checks included calibration and alignment of the Biomek 2000 performed monthly. Mass accuracy, resolution and sensitivity of the spectrometer were evaluated monthly using the insulin standard chip and the bovine IgG standard chip (Ciphergen). A normal phase ProteinChip, NP20 (Ciphergen) was run weekly, loaded with All-in-1 Protein standard II for external calibration of the spectra. To minimize slight systematic shifts in the time-of-flight data from one spot to another one, we used the CE to calculate a spot-to-spot correction factor. The correction factor was calculated from 8 spectra (One spectrum per spot position) of All-in-I Peptide Standard (Ciphergen) on NP20 ProteinChip.

### Data Analysis

All data were analyzed in CE. We applied baseline smoothing before fitting the baseline using a moving average filter window of 25 points, and an automatic fitting width. We used an average filter of 0.2 times expected peak width, to remove high frequency noise from the spectrum improving the S/N. Spectral intensities were normalized by total ion current and spectra with normalization factor > 2SD were excluded. The spectra were calibrated from a weighted 3 parameter quadratic equation calculated from 4 protein standards (mass range 7 to 30 kDa). Prior to alignment we did peak detection with settings of peak height and valley depth at 6 times the noise. Peak alignment was performed using the following settings: 0.2% of mass window and minimum S/N of 5. Peaks were identified using the CE Biomarker Analysis Module Cluster Wizard according to these settings: first pass S/N ≥ 3 and valley depth ≥ 3, minimum peak threshold 80% of all spectra, preserving all 1^st ^pass peaks, mass window 0.2% of mass, second pass S/N ≥ 2 and valley depth ≥ 2, add estimated peaks to complete clusters, autocentroid, and m/z range 3000–30000.

Calculations of average CVs for peak intensity were accomplished in Microsoft Excel. Statistical analyses using a non-parametric Kruskal-Wallis test, with a bootstrap of 2000 randomizations for multiple test correction, were performed using Partek Genomics Suite (version 6.2 Copyright ^© ^2006).

## Competing interests

The author(s) declare that they have no competing interests.

## Authors' contributions

DDR was responsible for the laboratory studies and data acquisition. She performed some of the data analysis and aided in the writing of the manuscript. TW designed the study, performed the statistical analysis and helped write the manuscript. SDV was responsible for critical assessment and revision of the manuscript. The findings and conclusions in this report are those of the authors and do not necessarily represent the views of the funding agency. All authors have read and approve the final manuscript.
